# Effects of Membrane Structure on Oil–Water Separation by Smoothed Particle Hydrodynamics

**DOI:** 10.3390/membranes12040387

**Published:** 2022-03-31

**Authors:** Jie Liu, Xiaoping Xie, Qingbang Meng, Shuyu Sun

**Affiliations:** 1 Computational Transport Phenomena Laboratory, Physical Science and Engineering Division (PSE), King Abdullah University of Science and Technology (KAUST), Thuwal 23955-6900, Saudi Arabia; jie.liu.1@kaust.edu.sa; 2 School of Mathematics, Sichuan University, Chengdu 610064, China; xpxie@scu.edu.cn; 3 Institute of Geophysics and Geomatics, China University of Geosciences, Wuhan 430074, China; mengqb@cug.edu.cn

**Keywords:** membrane, oil–water separation, smoothed particle hydrodynamics

## Abstract

Membrane has been considered an effective tool for oil–water separation. By using the smoothed particle hydrodynamics (SPH) method, the effects of membrane structure on fluid separation were studied thoroughly in this paper. The oil–water two-phase fluid was generated as particles, while the membrane was built with solid particles, which was able to select the fluid particles. In general, the developed SPH method in this paper can evaluate separation performance with different membrane shapes, pore size distributions, membrane thickness and fluid properties. We suggest to the industry a potential approach to promote separation based on our simulation results, including adding the external force in the selected direction and demulsification for the bulk phase liquid particles. The triangular membrane performs well with the conditions for various parameters, as a result of its insensitivity to inhibiting factors. The effectiveness and robustness of the proposed SPH scheme was validated by a number of numerical experiments, and we assessed the optimized membrane structure and operation manners in order to improve separation efficiency and long-term safety.

## 1. Introduction

In the past decades, increasing attention has been paid to the treatment of oil–water separation in the energy industry as water injection has been the main enhanced oil recovery (EOR) approach in many oil fields in the middle and late stages of development [1,2,3]. Numerical simulation of multiphase fluid flow problems is a popular and effective approach in the study of oil–water separation [4], and research has studied the flow behavior of discretized meshes [5,6,7,8,9,10,11,12]. Conventional mesh methods can handle the multiphase flow simulation by meshing the computational domain [13,14,15], but for fluid flow with a free surface, which emerges as the initial state of membrane separation, the Lagrange method can prove a good option in the case of large deformation [16].

Membrane has been considered an effective tool in oil–water separation in comparison with conventional methods, including physical and chemical separation approaches [17,18,19]. The membrane separation has three main mechanisms: adsorption, sieving and electrostatic phenomenon [20]. The physical methods can be carried out using the processes of filtering, evaporation, and adsorption, which involves the consumption of active carbon, sand filter, resins and zeolites [21]. Chemical methods include the processes of electrochemical adsorption and oxidation [20], although pollution always emerges in the processes of chemical treatments. The membrane separation method has a relatively lower cost and environmental friendliness with regard to the treatment of waste water, compared with the previously mentioned conventional methods, but the separation efficiency can be further improved with optimization of the membrane structure and operation manners.

The traditional grid-based methods were developed by transferring parameters between neighboring meshes, such as the finite element method, finite volume method and lattice Boltzmann method [22], which had not been applied on fluid–membrane interaction dynamics. The SPH method was developed by discretizing the computational domain into particles, which are then applied to simulate the oil–water separation and evaluate the separation performance under various circumstances to optimize the membrane structure and operation manners.

SPH was proposed by Lucy and Monaghan [23,24] as a meshless Lagrange method, and it was proved to be good at handling problems with large deformation and free surfaces. Thus, the SPH method has been successful in many engineering applications, including astrophysics, ship movement, debris flow and fluid–solid interaction [25,26,27,28], such as wave–structure interaction [29,30,31]. The SPH method is generally developed based on two approximations: kernel function approximation and particle approximation. There are two types of SPH method, mainly developed by fluid simulation: incompressible SPH (ISPH) and weakly compressible SPH (WSPH) [32,33]. In this work, we used WSPH, which solved the equation of state for pressure calculation.

In this work, the oil–water membrane separation was carried out using the SPH method. The flow abilities of various membrane structures were tested, involving the width and length of channels as well as membrane shapes. The properties of the oil phases were also examined to investigate the effects on the separation, realized by different droplet sizes and viscosities. The external force and the oil–water circumstances were studied for the dynamic phase behaviors.

## 2. Governing Equation and SPH Model

### 2.1. Governing Equation

In this work, the oil and water phases were discretized in the SPH forms [34,35]. all the symbols were listed and explained in Table 1. The mass and momentum conservation equations in Lagrange form were employed to describe the fluid flow [36,37], and the equation of state was adopted to close the governing equations [38],

(1) $$\frac{d\rho }{dt}=-\rho \nabla \cdot u$$

(2) $$\rho \frac{du}{dt}=-\nabla p+\nabla \cdot \left(\mu \left(\nabla u+\nabla u^{T}\right)\right)+g+F^{S}$$

(3) $$p=p_{eq}\frac{n}{n_{eq}}$$

where ρ denotes the density of oil and water phases; u denotes fluid velocity; **g** denotes the gravity acceleration; **F***^S^* denotes the surface tension force; *p_eq_* denotes the equilibrium pressure; *n_eq_* denotes the equilibrium number density; and *p* and *n* denote the system pressure and number density. The two-phase boundary condition was controlled by the Young–Laplace equation [39,40,41],

(4) $$\left(p_{o}-p_{w}\right)n=\left(\tau_{o}-\tau_{w}\right)\cdot n+k\sigma n$$

(5) $$\sigma_{wo}\cos\theta_{e}+\sigma_{so}=\sigma_{sw}$$

where $p_{o}$ and $p_{w}$ denote the pressures of oil and water phases, respectively; $\tau_{o}$ and $\tau_{w}$ denote the viscous stress tensors of oil and water phases, respectively; n is the normal unit vector perpendicular to the interface between oil and water phases; $\sigma_{wo}$ denotes the surface tension coefficient between water and oil phases; $\sigma_{so}$ and $\sigma_{sw}$ are oil–water surface coefficients, respectively; and $\theta_{e}$ denotes the equilibrium contact angle.

**Table 1:** Table of notation.

Symbol	Description
ρ	The density of oil and water phases
t	Time
u	The fluid velocity
**F** ^*S*^	The surface tension force
**g**	The gravity acceleration
*p_eq_*	The equilibrium pressure
*n_eq_*	The equilibrium number density
*p*	The system pressure
*n*	The number density
$p_{o}$	The pressures of oil phase
$p_{w}$	The pressures of water phase
$\tau_{o}$	The viscous stress tensor of oil phase
$\tau_{w}$	The viscous stress tensor of water phase
$\sigma_{so}$	The oil surface coefficient
$\sigma_{so}$	The water surface coefficient
$\theta_{e}$	The equilibrium contact angle
A(r)	The field function
$m_{b}$	The mass function of particle *b*
$\rho_{b}$	The density function of particle *b*
$A_{b}$	The field function of particle *b*
*h*	The smooth length
r	The distance between two particles
*W*	The kernel function
$\sigma_{d}$	The kernel normalization factor in different dimensions
$s_{\alpha \beta }$	The strength coefficient of the interaction force between particle *a* in *α* phase and particle *b* in *β* phase
${\hat{n}}_{j}$	The unit normal vector on the solid particle j
$b_{j}$	The prescribed acceleration for solid particle
$N_{f}$	The number of fluid particles
$N_{bound}$	The number of solid particles

### 2.2. SPH Model

As a meshless method, the SPH method is applied on the equilibrium and dynamic fluid behaviors by discretizing the domain into particles [42,43],

(6) $$A\left(r\right)=\int^{\;}A\left(r^{\prime }\right)W\left(r-r^{\prime },h\right)dr^{\prime }$$

(7) $$A\left(r\right)=\underset{b}{\sum }m_{b}\frac{A_{b}}{\rho_{b}}A\left(r^{\prime }\right)W\left(r-r_{b},h\right)$$

where A(r) denotes the field function; $m_{b}$, $\rho_{b}$ and $A_{b}$ are the mass, density and field function of particle *b*; *h* denotes the smooth length; r denotes the distance between two particles within the range of the smooth length; and *W* is the kernel function to judge the weight of particles. In this work, to reduce the cost of computational resources and ensure high accuracy, we adopted the cubic spline function as the kernel function [42],

(8) $$W\left(r,h\right)=\sigma_{d}\{\begin{array}{c} 6\left(q^{3}-q^{2}\right)+1,\;\;\;\;0\leq q\leq 0.5\;\;\\ \;\;\;2{\left(1-q\right)}^{3},\;\;\;\;\;\;\;0.5<q\leq 1\;\;\;\\ 0,\;\;\;\;\;\;\;\;\;\;\;\;q>1\;\; \end{array}$$

where $q=\frac{\left\|r\right\|}{h}$ and $\sigma_{d}$ denotes the kernel normalization factor in different dimensions, $\sigma_{1}=\frac{4}{3h}$, $\sigma_{2}=\frac{40}{7\pi h^{2}}$ and $\sigma_{3}=\frac{8}{\pi h^{3}}$. Based on the Lagrange form of the SPH method, the continuity equation can be written as follows [44]:

(9) $$\frac{d\rho_{a}}{dt}=\underset{b}{\sum }m_{b}u_{ab}\cdot \nabla_{a}W_{ab}$$

where $u_{ab}=u_{a}-u_{b}$, $W_{ab}=W_{a}-W_{b}$, and $\nabla_{a}W_{ab}=-\nabla_{b}W_{ab}$. For the momentum conservation equation, the gradient of pressure needs to be calculated first. In order to obtain the symmetric form of the pressure gradient acceleration term, it is commonly written as follows:

(10) $$\left(\frac{1}{\rho }\nabla p\right)=\nabla \left(\frac{p}{\rho }\right)+\frac{p}{\rho^{2}}\nabla p$$

Change the pressure into SPH form and we can obtain [45]:

(11) $${\left(\frac{1}{\rho }\nabla p\right)}_{a}=\underset{b}{\sum }m_{b}\left(\frac{p_{a}}{\rho_{a}^{2}}+\frac{p_{b}}{\rho_{b}^{2}}\right)\nabla_{a}W_{ab}$$

According to the research of Monaghan [46], it can be written as:

(12) $${\left(\frac{1}{\rho }\nabla p\right)}_{a}=\underset{b}{\sum }m_{b}\left(\frac{p_{a}+p_{b}}{\rho_{a}\rho_{b}}\right)\nabla_{a}W_{ab}$$

The second term of the momentum conservation equation is the viscosity term; in this work, the artificial viscosity was adopted, developed by the first derivative of SPH and finite difference approximation [44], commonly in the following form [47,48]:

(13) $${\left(\frac{\mu }{\rho }\nabla^{2}u\right)}_{a}=\underset{b}{\sum }m_{b}\frac{\left(\mu_{a}+\mu_{b}\right)r_{ab}\cdot \nabla_{a}W_{ab}}{\rho_{a}\rho_{b}\left(r_{ab}^{2}+0.01h^{2}\right)}u_{ab}$$

The third term is the surface tension, which is caused by the interaction forces of two different phases. In SPH, it is considered as the pairwise force between two particles which are in two phases [49]. Similar to the molecular dynamics theory, it shows a strong repulsive force if two particles are too close; otherwise, it performs as an attractive force, and the interaction force reaches zero if the distance exceeds the smooth length [50,51,52]:

(14) $$F_{ab}^{s}=\{\begin{array}{c} -s_{\alpha \beta }r_{ab}\left[A\Psi_{\varepsilon_{0}}\left(r_{ab}\right)+\Psi_{\varepsilon }\left(r_{ab}\right)\right]\\ 0 \end{array}\;\;\;\;\begin{array}{c} r_{ab}\leq h\\ r_{ab}>h \end{array}\;\;$$

where $s_{\alpha \beta }$ denotes the strength coefficient of the interaction force between particle *a* in *α* phase and particle *b* in *β* phase. $\varepsilon =\frac{h}{3.5}$, $\varepsilon_{0}=\frac{\varepsilon }{2}$, $\Psi_{\varepsilon }\left(r_{ab}\right)=e^{\frac{r_{ab}^{2}}{2\varepsilon^{2}}}$ and $A={\left(\frac{\varepsilon }{\varepsilon_{0}}\right)}^{3}$ in two spatial dimensions. In order to achieve the immiscible phase behavior between two phases, Alexandre et al. [39,49] set the $s_{\alpha \alpha }>s_{\alpha \beta }$ and $s_{\beta \beta }>s_{\alpha \beta }$, and derived the form of $s_{\alpha \beta }$ as follows:

(15) $$\{\begin{array}{c} s_{\alpha \alpha }=s_{\beta \beta }=0.5n^{-2}{\left(\frac{h}{3}\right)}^{-5}\frac{\sigma }{\lambda }\;\\ s_{s\alpha }=0.5n^{-2}{\left(\frac{h}{3}\right)}^{-5}\frac{\sigma }{\lambda }\left(1+0.5\cos\theta \right)\\ s_{s\beta }=0.5n^{-2}{\left(\frac{h}{3}\right)}^{-5}\frac{\sigma }{\lambda }\left(1-0.5\cos\theta \right) \end{array}\;\;$$

where n denotes the average number density of particles; σ denotes the surface tension coefficient; θ is the contact angle; λ is a constant; and $\lambda =\frac{3}{4\pi^{2}}\left(2^{7}-3^{2}\times 2^{4}\pi^{2}+3^{3}\pi^{4}\right)$. Therefore, we can obtain the momentum conservation equation:

(16) $$\frac{du}{dt}=-\underset{b}{\sum }m_{b}\left(\frac{p_{a}+p_{b}}{\rho_{a}\rho_{b}}\right)\nabla_{a}W_{ab}+\underset{b}{\sum }m_{b}\frac{\left(\mu_{a}+\mu_{b}\right)r_{ab}\cdot \nabla_{a}W_{ab}}{\rho_{a}\rho_{b}\left(r_{ab}^{2}+0.01h^{2}\right)}u_{ab}+g+F^{s}$$

For the oil and water two-phase flow via membrane, the flow channel is so small that it induces instability in the SPH calculation, while it is more stable when considering the pairwise interaction force [33,49]. For the interaction forced by a solid wall, according to previous studies [32,33], we can obtain the boundary condition as follows:

(17) $$F_{i}^{bound}=\overset{N_{bound}}{\underset{j=1}{\sum }}f_{ij}^{bound}$$

(18) $$f_{ij}^{bound}=\{\begin{array}{c} -\left[U_{max}^{2}\frac{\min\left(\left(u_{i}-u_{j}\right)\cdot {\hat{n}}_{j},-1\right)W_{ij}H_{ij}{\hat{n}}_{j}}{\left|r_{ij}\cdot n_{j}\right|}\right],\;\;\left(u_{i}-u_{j}\right)\cdot {\hat{n}}_{j}<0\\ 0,\;\;\left(u_{i}-u_{j}\right)\cdot {\hat{n}}_{j}>0 \end{array}$$

where the *i* and *j* denote the fluid and solid particles, and the $u_{i}$ and $u_{j}$ denote the fluid velocity and solid velocity. The ${\hat{n}}_{j}$ denotes the unit normal vector on the solid particle *j*. The velocity and pressure of solid particles can be calculated as follows:

(19) $$u_{j}=-\frac{\sum_{i}^{N_{f}}u_{i}W_{ij}}{\sum_{i}^{N_{f}}W_{ij}}$$

(20) $$p_{j}=\frac{\sum_{i}^{N_{f}}p_{i}W_{ij}+\left(g-b_{j}\right)\cdot \sum_{i}^{N_{f}}\rho_{i}r_{ij}W_{ij}}{\sum_{i}^{N_{f}}W_{ij}}$$

where the $N_{f}$ and $N_{bound}$ denote the number of fluid and solid particles, and the $b_{j}$ denotes the prescribed acceleration for solid particles.

## 3. Results and Discussion

In this paper, the oil–water separation was carried out using the SPH method. The simulation schemes are validated in Section 3.1. The effects of membrane structure, such as the shape, pore size and thickness of membrane, are examined in Section 3.2. In Section 3.3, we report the tests on the properties of the oil phase, such as oil viscosity and the size of the oil droplet, on the separation behaviors. Finally, we analyze the external conditions in Section 3.4.

### 3.1. Scheme Validation

**Figure 1:**
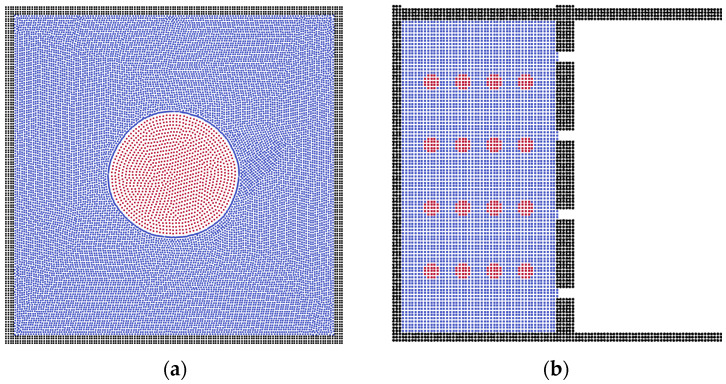
(**a**) The two-phase validation model and (**b**) the physical model of oil–water separation. Black particles represent the solid wall and membrane, blue particles represent the water phase, and red particles represent the oil phase.

By utilizing the SPH method, we calculated the oil–water separation via flat membrane. The fluid model was constructed with sixteen oil droplets and an aquifer environment, which was driven by a constant acceleration force. The two-phase validation model and separation model are shown in Figure 1, where the red and blue particles are built as the oil and water phases, respectively, and the black particles represent the solid wall.

**Figure 2:**
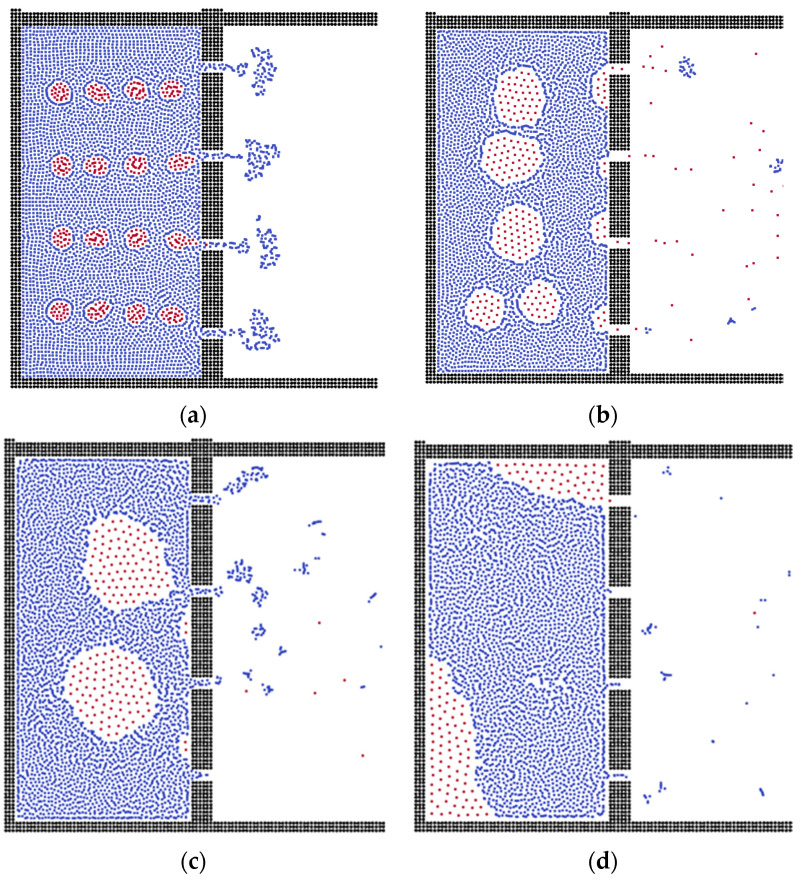
The separation processes during different time steps. (**a**) Step = 5, (**b**) Step = 20, (**c**) Step = 100, (**d**) Step = 200.

Because of the effect of surface tension, the oil-in-water state commonly performs as the sphere droplet, and the shape of the oil droplets in water is adopted. In the first fifteen steps, the oil droplets move to the membrane pores gradually, as shown in Figure 2, during which process the oil droplets combine together to form bigger oil clusters, making it harder to flow through the membrane channels.

As can be seen in Figure 2, after 30 steps, the oil clusters accumulate on the surface of the membrane due to the surface tension. In the flat membrane system, the oil droplets can pass through the membrane easily, and the oil particles, which cannot be further excluded, adsorb on the corner of the box. Therefore, the flat membrane can separate small-size oil droplets, which agrees well with previous work [53]. When the droplets gather into bigger oil clusters, the membrane becomes blocked, but this shows a good separation boundary within the waste water domain [54]; afterwards, the oil clusters move and adsorb on the corner of the system due to the lower surface energy.

### 3.2. The Effects of Membrane Structures

**Figure 3:**
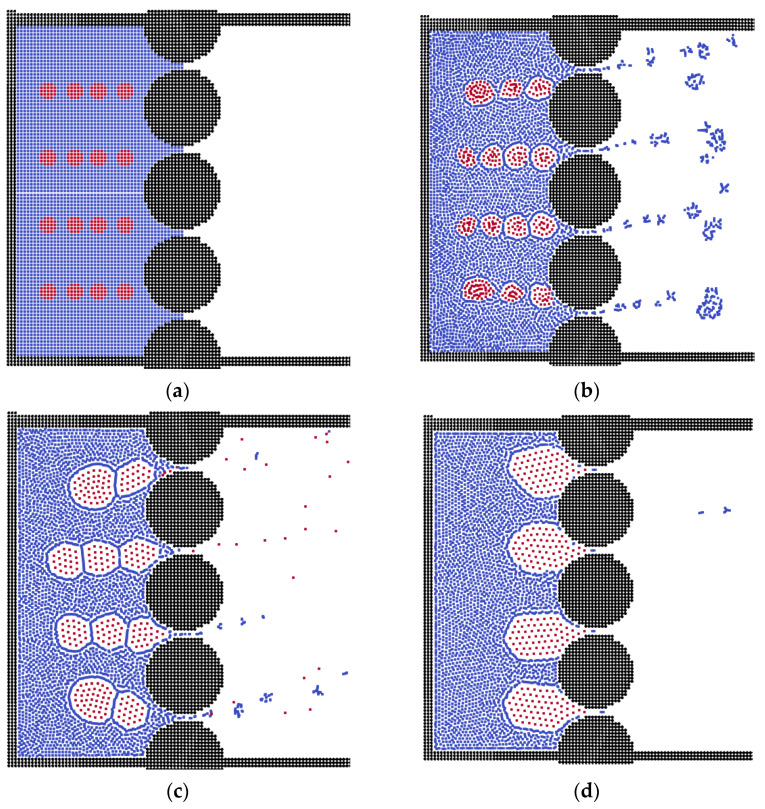
The separation processes in the sphere membrane during different time steps. (**a**) Step = 0, (**b**) Step = 20, (**c**) Step = 100, (**d**) Step = 200.

Different polymeric membranes have been used for oil–water separation [55], but in this part of the study, in order to examine the effects of membrane structure, membranes in spherical and triangular shape were constructed. Figure 3 demonstrates the processes of separation in different time steps. Initially, the small oil droplets move to the membrane channels, as in previous results, and some small oil droplets pass the channels before changing into bigger clusters. However, the channel size is so small that the small oil droplets combine together quickly before flowing out. Finally, the oil clusters are formed and block the channels [56]. This is because the sphere shape has a larger contact area with the oil clusters, suggesting that it induces less surface energy for the oil phase. Thus, the channels are blocked totally by oil clusters.

**Figure 4:**
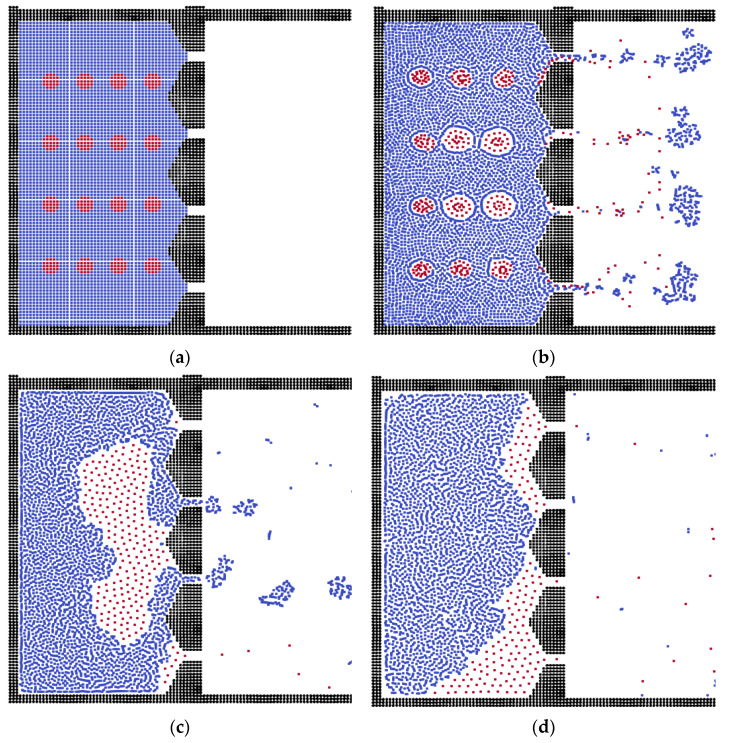
The separation processes in the triangular membrane during different time steps. (**a**) Step = 0, (**b**) Step = 20, (**c**) Step = 100, (**d**) Step = 200.

For the triangular membrane structure, the separation result is similar to that of the sphere membrane system. The difference emerges in the final stage: the oil phase forms the oil layer on the membrane surface, stopping further fluid flow, as shown in Figure 4. The reason is that the contact area is controlled by the shape of the membrane. If the shape of the membrane facilitates the connectivity of oil clusters, the oil phase will form an oil layer to block the membrane; otherwise, they will block the channels independently.

**Figure 5:**
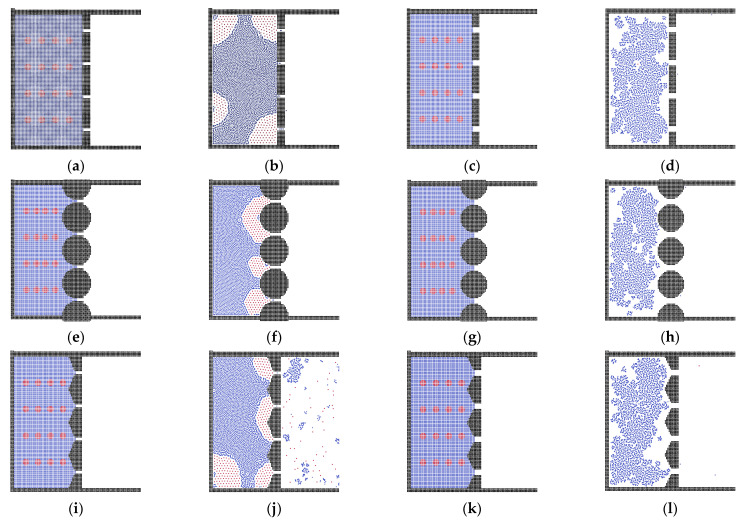
The results with different channel sizes; (**a**,**b**,**e**,**f**,**i**,**j**) with channel width of 12 μm; (**c**,**d**,**g**,**h**,**k**,**l**) and with channel width of 20 μm.

To investigate the effect of channel width, as shown in Figure 5, we set models with different channel sizes. In the flat membrane system, the smaller channels hold more oil particles in the box, while in the membrane with a wider channel, the entire oil phase is pushed out. This is the same in the sphere and triangular membrane systems. However, in the small-size channel system, the sphere and triangular membranes block the oil phase, because the small pore size results in a stronger interaction between fluid particles and pores.

**Figure 6:**
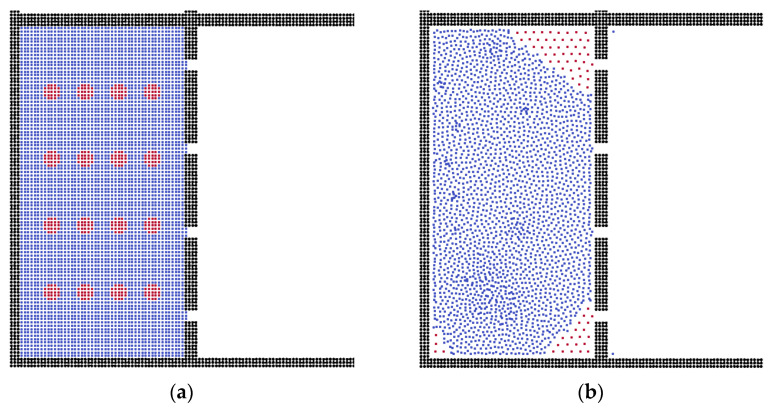

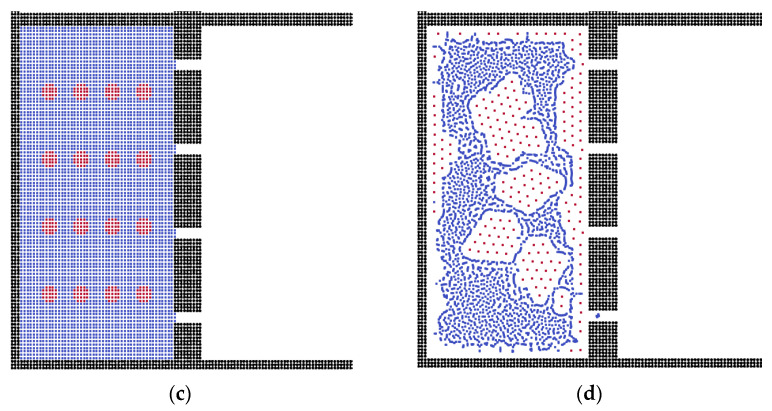
Results of separation with different thicknesses of membrane, (**a**,**c**) are the initial conditions, thickness = 10 and 30 μm, respectively; (**b**,**d**) are the results.

The fluid flow was affected by the channel surface, and we calculated different models in order to explain the effects of membrane thickness. For the thin membrane structure, as shown in Figure 6, oil particles adsorb on the corner, which is similar to that previously discussed. However, using a thicker membrane structure, Figure 6d shows more residual oil particles, and the structure of the oil phase tends to form a layer. This is because the thicker membrane induces the stronger interaction force between fluid and solid wall particles.

### 3.3. Oil Properties on the Separation Behavior

**Figure 7:**
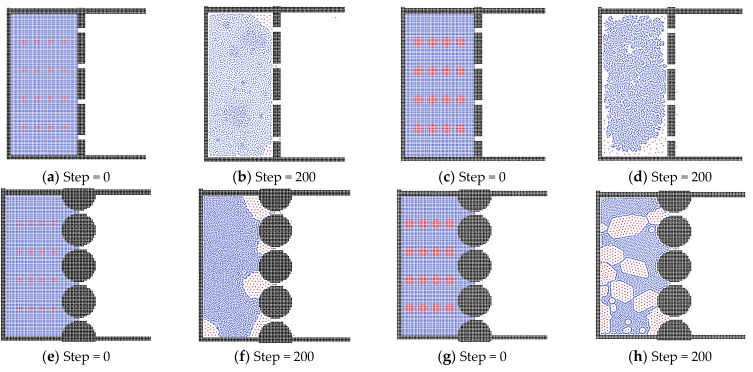

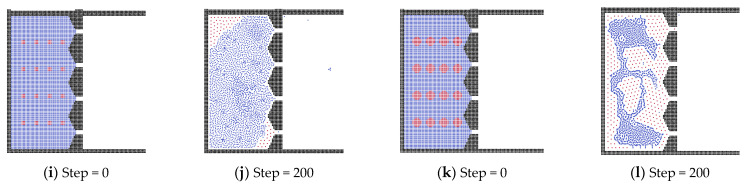
The different oil droplet size systems in membrane for the oil–water separation, (**a**,**b**,**e**,**f**,**i**,**j**) forming oil droplets with radii of 4 μm; (**c**,**d**,**g**,**h**,**k**,**l**) forming oil droplets with radii of 8 μm.

To investigate the effect of oil droplet size, we studied the oil droplets with different radii in three shapes of membrane model, as can be seen in Figure 7. Apart from the oil size and the membrane structure, other parameters were kept constant, such as the width of channel, thickness of membrane, viscosity of oil phase and acceleration force. In the flat membrane system, obviously, the size of oil droplets only affected the quantity of residual oil particles. In the sphere membrane system, the small oil droplets can pass the channel easily, meaning they have to accumulate into bigger oil clusters to block the system, as shown in Figure 7f. But for the bigger oil droplets, they do not need to be accumulated into clusters, because the channels only need one droplet to be blocked, as can be seen in Figure 7h. For the triangular membrane system, the channel cannot be blocked by small oil droplets, even if they are merged. But for the bigger oil droplets, they can totally block the membrane channels, performing as the oil layer around the box, and the liquid state is changed to water in oil.

**Figure 8:**
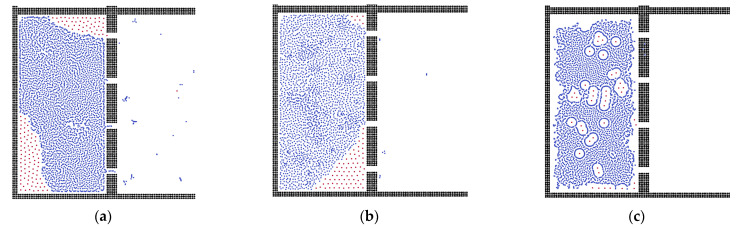
Results of separation with different oil viscosities: (**a**) 0.2 mPa·s; (**b**) 0.4 mPa·s; (**c**) 0.8 mPa·s.

To examine the effect of oil viscosity, as shown in Figure 8, oil droplets with different viscosities were examined. As we discussed, the oil particles would remain in the corner of the system because there was less surface energy. Thus, the residual oil particles always stayed in the same place. In the membrane system with higher oil viscosity, the oil particles tended to adsorb on the membrane surface.

### 3.4. Analysis of External Force and State of Liquid

**Figure 9:**
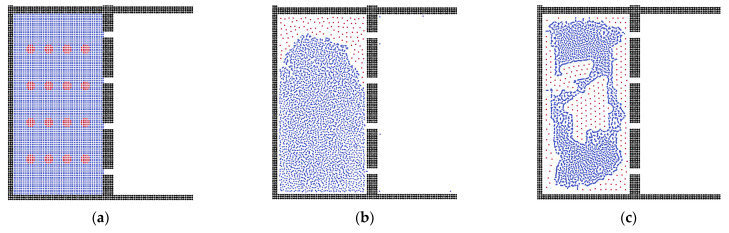

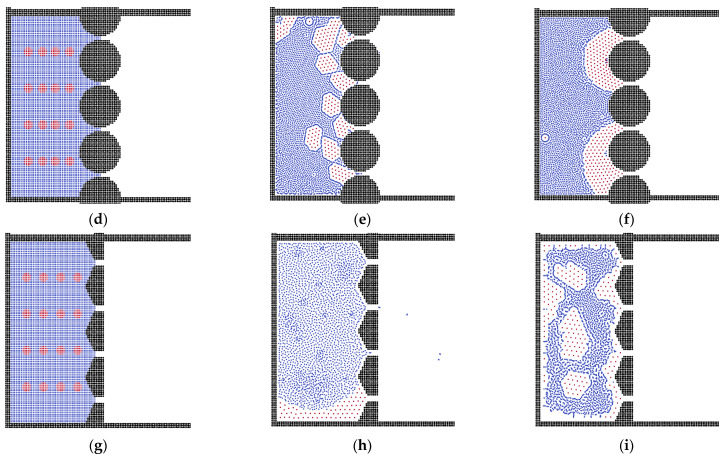
Results with different driving forces: (**a**,**d**,**g**) initial conditions of three different shape membrane systems; (**b**,**e**,**h**) controlled with 0.0005 m·s^−2^; and (**c**,**f**,**i**) controlled with 0.0015 m·s^−2^.

As shown in Figure 9, in the flat membrane system, there are more residual oil particles in the box under the condition of a smaller driving force. If we used a stronger external force on the fluid particles, the oil particles accumulated on the membrane surface quickly, blocking the channels. In the sphere membrane system, the oil droplets in both models blocked the channels. The difference is that the stronger driving force led to bigger oil clusters on the membrane surface. In the model of triangular membrane, the results are similar to those of the flat model, but there are fewer oil particles due to its conductivity.

**Figure 10:**
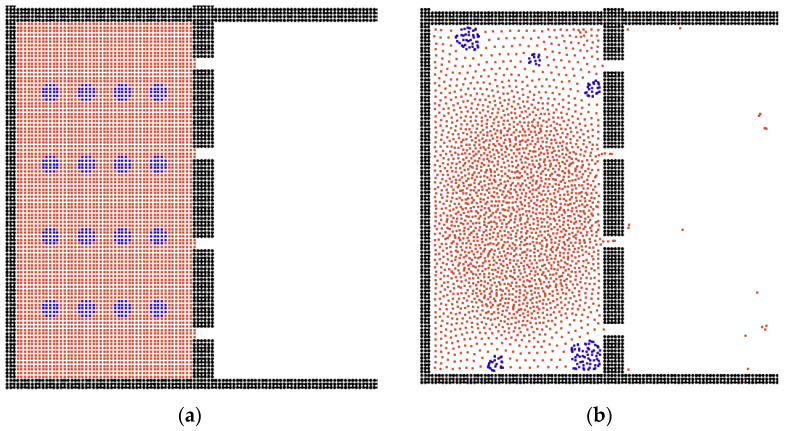
**(a)** The initial condition of water in oil. (**b**) The separation result for water in oil system. The red particles represent the oil phase and the blue particles represent the water phase.

As shown in Figure 10, we also studied the influence of oil–water state behaviors. If we replace the oil droplets in the water phase into water droplets in the oil phase, the water particles cannot go through the channel easily, because the surface tension stops them from getting out of the box. Here, the oil phase particles can pass freely through the channels compared with the oil-in-water system. Finally, the effects of different parameters on the oil–water separation are summarized in Table 2, and we demonstrated that the triangular membrane was the better choice.

## 4. Conclusions

In this work, we studied oil–water separation in membrane systems, using the SPH method. Compared with bulk phase particles, due to the effect of surface tension, the oil droplets showed stronger resistance in the processes of passing the membrane channels. With regard to the shape of membranes, the flat membrane structure showed less selectivity for water and oil. The sphere membrane facilitated accumulation of the oil phase, due to its larger contact area with oil phase. Different from the low selectivity in the flat membrane and excessive selectivity in the sphere membrane, the triangular membrane structure proved good for accumulation with a better conductivity. The flow ability demonstrated a positive relationship with the width of channel, while it was negative for the thickness of the membrane. By increasing the external force on the fluid particles, the flow ability of liquid was able to be improved, but it also induced the quick accumulation of oil particles and blocked the channels accordingly. Higher oil viscosity led to the oil layer on the membrane surface, preventing the oil–water separation, though it promoted the accumulation of the oil phase. The smaller oil droplets always showed a good passing ability, while bigger oil droplets tended to form clusters and adsorb on the membrane surface, especially for the sphere and triangular membranes. Considering the different effects, the triangular membrane is the better choice because it is insensitive to the inhibiting factors. This work provides a comprehensive study of the effects of various membrane structures on oil–water separation, although we do not consider the effect of electrostatic behavior, which may be studied in our future research.

## Figures and Tables

**Table 2:** Evaluation of oil–water separation with different parameters.

	Pore Size	Thickness	Droplet Size	Viscosity	External Force
Flat	↑	↓	↑	↓	↑
Triangular	↑	↔	↔	↓	↑
Sphere	↑	↔	↓	↓	↑

↑: Facilitation, ↓: Inhibition, ↔: Insensitive.

## Data Availability

Not applicable.
